# 

**Published:** 1885-09

**Authors:** 


CONTENTS.

Vol. 32 No. 9

| | PAGE. | | PAGE. |
|---|---|---|---|
| Erysipelas | 1 | Vivisection Indispensable to the Progress of Medicine | 10 |
| Toads as a Tonic | 2 | Die'ary for the Diabetic | 11 |
| The Air and the Telescope | 2 | Eating in the Cars | 11 |
| Must Keep up with the Times | 3 | Influences | 12 |
| A Domestic Secret | 3 | Signatures | 12 |
| Carbolic Acid | 5 | The Mind | 13 |
| Asthma | 6 | Exercise | 15 |
| Fever and Ague | 7 | Poisoning Children | 17 |
| Tomatoes as Food | 7 | Checked Perspiration | 18 |
| Suns:roke, or Thermic Fever | 8 | Miscellaneous | 20 |
| Sun Stroke | 9 | | |

